# Excessive internet use among Finnish young people between 2017 and 2021 and the effect of COVID-19

**DOI:** 10.1007/s00127-024-02723-0

**Published:** 2024-07-10

**Authors:** Olli Kiviruusu

**Affiliations:** Mental Health Team, Department of Public Health and Welfare, Finnish Institute for Health and Welfare, P.O. Box 30, Helsinki, 00271 Finland

**Keywords:** Excessive Internet use, Internet addiction, COVID-19, Adolescent, Depression, Anxiety

## Abstract

**Purpose:**

An increase in excessive Internet use (EIU) among adolescents during the COVID-19 pandemic was suggested in many studies. However, these studies were mostly based on cross-sectional and/or unrepresentative samples.

**Methods:**

Using data from a nationwide Finnish school survey in the years 2017, 2019 and 2021 (*N* = 450,864; aged 13–20 years), changes in the prevalence of EIU (EIUS, 5-item) were assessed. The effects of COVID-19 (year 2021 vs. 2017/2019 combined) and linear trend were analyzed in logistic regression models. Models were adjusted for loneliness, depression, anxiety, and sociodemographic factors.

**Results:**

Among males, EIU prevalence varied minimally (7.8–8.1%) from 2017 to 2021. Among females, the prevalence increased from 6.8 to 11.7% and the effect of COVID-19 was significant (OR = 1.53; *p* < 0.001). Including the linear trend in the model turned the COVID-19 effect on EIU among females non-significant (*p* = 0.625), whereas the trend was significant (OR = 1.17; *p* < 0.001). Adjusting the models with mental health-related factors attenuated the effect of COVID-19 to some extent, but not the effect of linear trend.

**Conclusions:**

There is a sex difference in the way the prevalence of EIU developed from 2017 to 2021 among Finnish adolescents. In males, there was no indication of increased prevalence of EIU and among females, while the effect of COVID-19 was first found, it was also suggested to be a product of a trend already started before the COVID-19 pandemic. These results are in contrast with some earlier studies suggesting an effect of COVID-19 on EIU.

**Supplementary Information:**

The online version contains supplementary material available at 10.1007/s00127-024-02723-0.

## Introduction

In the wake of the technological innovations and advancements, the Internet has become an ever more integral part of our everyday lives, including school, work, and leisure time. Especially among adolescents and young adults, use of Internet and social media are common practices for social interaction and entertainment. While Internet use is beneficial most of the time and for most of the users, research evidence has accumulated to also suggest that excessive use of Internet, social media, and gaming are linked to negative outcomes including cognitive deficits and problems in physical and mental health [1–3]. And for some, the overuse of Internet may lead to the development of addictive type behaviors.

Excessive Internet use (EIU) (other commonly used terms include problematic or pathological Internet use, and Internet addiction (IA)) can be perceived as a behavioral addiction [4, 5]. In behavioral addictions, first introduced to the DSM-5 [6], the problematic behaviors evolve to have dependence-like symptoms with negative consequences on everyday functioning, while no ingestion of a psychoactive substance is included. Essential criteria to EIU as an addictive behavior have been suggested to include (1) salient, excessive Internet use associated with preoccupations and neglect of basic needs, (2) withdrawal symptoms when Internet is not accessible, (3) tolerance and need for more hours of use, (4) relapses when trying to control the behavior, and (5) adverse consequences and conflicts in key areas of life such as interpersonal relationships and vocational and educational achievement [4, 7]. EIU covers a wide range of problematic behaviors, including excessive social media use, gaming, gambling, streaming, viewing of pornography, et cetera. For some of these more specific behaviors (i.e., gaming and gambling) there is a corresponding diagnosis in the diagnostic systems, but for the more generic categories (i.e., EIU or IA), no diagnosis appears in any official diagnostic system.

The prevalence rates of EIU/IA vary widely depending on sample characteristics, study designs and the used measures to detect excessive use of Internet. In a recent meta-analysis covering the years 2003–2018 with a total of 693,306 participants, Pan et al. [8] found a pooled prevalence of 7.0% for generalized IA, which was considerably larger than the prevalence of Internet gaming disorder (2.5%). Importantly, they found a clear increasing trend of IA with more recent studies reporting higher prevalence [8]. Regarding younger age groups, the same increasing trend has also been reported in college students [9]. More generally, young age and male sex seem to be associated with EIU [10]. Pre-pandemic prevalence among adolescent and young adult samples ranged from 8.4% up to over 30% levels as reported in some recent reviews [11, 12]. Among young males, the prevalence of EIU has been reported to be higher than rates found among females [9, 13]. However, Su et al. [14] reported in their meta-analysis that, while the prevalence of Internet gaming disorder is higher among males than females, the opposite is true for social media addiction. Results from a recent U.S. study indicate that these sex differences are likely to exist among adolescents as well [15].

During the COVID-19 pandemic, there was an increased worry about the negative effects of the lockdowns and “stay at home” restrictions on problematic Internet usage patterns [16]. As the use of Internet increased during the pandemic, it was thought that the related negative consequences of Internet use would increase as well [17]. Especially of concern were adolescents and students, among whom use of Internet was a very prominent feature of everyday life to begin with, and who now were faced with school closures, remote education, and restrictions on leisure time activities—all likely to further exacerbate their Internet use. And indeed, there were many reports (mainly among adolescents and young people) from the early days of the pandemic suggesting increases in prevalence of EIU/IA [11, 17, 18]. However, some caution remained warranted as to the claims of pandemic-related effects, since the majority of these studies were based on cross-sectional designs and relatively small or unrepresentative samples [11]. One rare exception to this was a Japanese longitudinal study by Oka et al. [19] reporting the prevalence of probable problematic Internet use increasing by 1.6 times during the pandemic (from December 2019 to July 2020) among both adults and young people. In another Japanese study of three consecutive cross-sectional school surveys, only a slight and non-significant increase of problematic Internet use from 4.6 to 5.2% between 2018 and 2020 was observed among 12–13-year-old adolescents [20].

Restrictions and school closures imposed during the pandemic caused social isolation and loneliness [21, 22]. Similarly, increased levels of depression and anxiety were reported, especially among adolescents, and especially among females [22, 23]. Both difficulties in social interactions and loneliness as well as mental health problems have been shown to be associated with EIU [3, 24, 25]. While the associations of EIU with loneliness, depression and anxiety are likely bi-directional [3], to the extent that these mental health-related factors do predispose one to EIU, increases in them would also have had an increasing effect on the EIU prevalence during the COVID-19 pandemic. Thus, their role needs to be considered when assessing the effect of the pandemic on the prevalence of EIU. Furthermore, some studies have indicated that the associations between problematic smartphone or social media use and psychological distress had even heightened during the pandemic [26].

As noted above, the majority of the studies claiming increases in EIU or AI during the pandemic were based on small or unrepresentative samples. Most of them were cross-sectional in design with no similar measurements from the pre-pandemic era. Studies where the pre-pandemic trends have been included in the analyses are even more scarce or non-existent. In the present study, using large nationwide school survey data from the years 2017, 2019 and 2021 with over 450,000 participants in total, changes in the prevalence of EIU were assessed among Finnish adolescents with a special interest on whether the changes were related to the COVID-19 pandemic or best conceptualized otherwise. Also, sex and age group differences were addressed and the role of contemporaneous developments in loneliness, depression, and anxiety during the study period.

## Methods

### Subjects

The data was obtained through the School Health Promotion (SHP) study, a nationwide classroom survey conducted every other year by the Finnish Institute for Health and Welfare (THL) [27]. The survey is based on total sampling, and gathers data on well-being, health, and life circumstances of 8th and 9th grade students in the comprehensive schools, and 1st and 2nd year students in general upper secondary schools and vocational education institutions. The students complete the SHP questionnaire independently and anonymously during a school lesson. They are informed about the study and the voluntary nature of participation. Guardians of students under 15 years old are informed in advance, and they have an option to decline for their child to participate. The institutional review board of THL has evaluated the SHP research plan [27].

The present study used data from the years 2017, 2019 and 2021, when the measure for EIU has been included in SHP. The coverage rate in 2021 was 75% among 8th and 9th graders, 71% in general upper secondary schools, and 32% in vocational education institutions [27]. In spring 2021, the SHP study was carried out at a time when Finland was facing the third wave of the pandemic (for more details see Supplementary Material).

### Measures

Excessive Internet use (EIU) was measured with the 5-item version of the Excessive Internet Use Scale (EIUS) [28]. The items cover five components of behavioral addictions [4, 29] with the statements: “I have tried spending less time online, but I have failed” (relapse); “I should spend more time with my family, friends or homework, but I spend all my time online” (conflict); “I have found that I was online even though I did not really feel like it” (tolerance); “I have felt anxious when I do not get online” (withdrawal symptoms); “I have failed to eat or sleep because of being online” (salience). The items were answered on a four-point scale (“never,” “not very often,” “fairly often,” “very often”) and the answers “fairly often” and “often” indicated a present symptom. The condition where the conflict symptom and at least three other symptoms were present was considered to indicate an increased risk of addictive behaviors [30, 31] labeled here as “excessive Internet use” (EIU).

Loneliness was asked with a simple question “Do you ever feel lonely?” The five answer categories were dichotomized to loneliness (“fairly often,” “all the time”) vs. no loneliness (“never,” “very rarely,” “sometimes,”). Depression was measured with the Patient Health Questionnaire-2 (PHQ-2), a 2-item self-reported screen shown to be a reliable tool among adolescents and adults to detect depression [32, 33]. The sum score (range 0–6) was dichotomized to depression (3 or more points) vs. no depression (< 3) [32, 33]. Anxiety was assessed using the 7-item Generalized Anxiety Disorder Scale (GAD-7) [34] proved to be a reliable and valid instrument for measuring self-reported generalized anxiety in the general population among adults [35] and adolescents [36]. The sum score (range 0–21) was dichotomized using the cut-off of 10 points or more to indicate cases with moderate to severe generalized anxiety symptoms [34].

The respondents reported their sex (male or female) and age. The mean age of the total sample was 15.8 (SD = 1.28) years. In the analyses, a three-category age group variable was used (Table 1). Sociodemographic factors used as control variables were family’s financial situation, living with both parents, origin, and geographical region (Table 1; Supplementary Material). The first three have been shown to correlate with EIU [37, 38]. In the present data, they had small but significant (*p* < 0.0001) correlations (Spearman rho) with EIU: family’s poor financial situation 0.04; living with both parents − 0.01; immigrant origin 0.07. Geographical region, based on the seven Regional State Administrative Agencies (see Table 1), was included in the control variables, as these agencies decided and administered many of the COVID-19-related restrictions, including restrictions on schools, leisure time activities, and hobbies. There were also small differences in EIU prevalence between regions (Chi^2^ = 94.38, df = 6, *p* < 0.0001).

**Table 1:** Frequencies of the study variables by study year, %

	Missing data %^a^		2017 *N* = 124,964^b^	2019 *N* = 146,603^b^	2021 *N* = 152,609^b^
**Sociodemographic and control variables**					
Sex	0.4/0.0				
Female			51.8	52.7	53.4
Male			48.2	47.3	46.6
Age group	0.4/0.0				
13–15 years old			42.8	45.1	46.0
16–17 years old			47.1	45.9	45.3
18–20 years old			10.1	9.0	8.7
Family’s financial situation	4.0/0.7				
Good			65.5	71.6	72.0
Moderate			27.1	22.6	22.9
Poor			7.4	5.8	5.1
Living with both parents	4.5/1.6		67.9	68.2	67.2
Origin	4.0/0.7				
Finnish-born parents			88.7	87.8	87.2
Multicultural family			6.7	7.2	7.4
Second-generation immigrant			1.8	2.0	2.2
First-generation immigrant			2.8	3.0	3.2
Region of Finland^c^	0.0/0.0				
Southern Finland			36.8	39.8	38.7
Southwestern Finland			13.3	12.5	12.4
Eastern Finland			10.5	9.7	9.8
Western and Inland Finland			24.4	23.8	24.0
Northern Finland			10.8	10.6	11.3
Lapland			3.4	3.0	3.2
Åland			0.7	0.6	0.6
**Mental health-related correlates**					
Loneliness	1.2/0.4		9.4	10.8	16.5
Depression	2.2/1.3		13.4	14.8	20.9
Generalized anxiety	1.1/0.3		11.6	12.6	19.5

^a^ Of all participants (before slash)/of those fulfilling the inclusion criteria for the study (after the slash)

^b^ Those fulfilling the inclusion criteria for the study (see methods)

^c^ Based on Regional State Administrative Agencies

### Statistical analyses

Analyses were done using IBM SPSS Statistics 28.0 software. Non-missing values were required for sex, age, and the EIU variable, resulting in the exclusion of 25,095 cases (5.6%) from the analyses, mainly due to missing information on EIU (5.0%). In addition, 1,593 (0.4%) cases were excluded due to implausible responding (see Supplementary Material) leaving 424,176 cases for the present analyses.

For prevalence, percentages and 95% confidence intervals (CI) of those with EIU were calculated (Table 2). Logistic regression was then used to analyze the effect of the COVID-19 pandemic on the prevalence of EIU (Table 3). First, the COVID-19 effect alone was analyzed using a dichotomous variable coded as “1” for the year 2021, otherwise “0”, thus contrasting the year 2021 against the two pre-pandemic survey rounds combined. Combining the years 2017 and 2019 was done to give a more reliable estimate of the pre-pandemic prevalence, not affected by yearly fluctuations. In the second phase, linear time parameter was added to the models. This second analysis models the effect of COVID-19 as the deviation between values observed in 2021 from values based on the linear trend during the study period. First, unadjusted logistic models were estimated separately for males and females and within sexes by age group (Table 3) and then adjusted models were fitted among males and females to assess whether sociodemographic and mental health correlates would have any effect on the development of EIU prevalence (Table 4). Sex differences in the effects were analyzed using sex × COVID-19 and sex × time interaction terms (Tables 3 and 4), and age group differences within the male and female models with age group × COVID-19 and age group × time interaction terms in the models (Table 3).

**Table 2:** Percentages and 95% confidence intervals (CI) of those with excessive Internet use in years 2017, 2019 and 2021 by sex and age group

	2017 *N* = 124,964		2019 *N* = 146,603		2021 *N* = 152,609
	% (95% CI)		% (95% CI)		% (95% CI)
Total	7.3 (7.1–7.4)		8.6 (8.4–8.7)		9.9 (9.8–10.1)
Males	7.8 (7.6–8.0)		8.1 (7.9–8.3)		8.0 (7.8–8.2)
Females	6.8 (6.6–7.0)		9.0 (8.8–9.2)		11.6 (11.4–11.8)
Males by age group					
13–15 years old	8.9 (8.6–9.3)		8.6 (8.3–8.9)		8.4 (8.1–8.7)
16–17 years old	6.9 (6.6–7.2)		7.6 (7.3–7.9)		7.7 (7.4–8.0)
18–20 years old	7.1 (6.4–7.7)		7.8 (7.1–8.5)		7.9 (7.2–8.5)
Females by age group					
13–15 years old	7.6 (7.3–7.9)		9.5 (9.2–9.8)		12.5 (12.2–12.9)
16–17 years old	6.3 (6.1–6.6)		8.5 (8.2–8.8)		10.9 (10.6–11.2)
18–20 years old	5.8 (5.2–6.4)		8.8 (8.1–9.4)		10.5 (9.8–11.2)

**Table 3:** Effects of the COVID-19 pandemic (year 2021 vs. 2017/2019) and linear time on the prevalence of excessive Internet use by sex and age group. Unadjusted models

	Model 1: COVID-19 only			Model 2: COVID-19 + linear time	
Subgroup	OR (95% CI)	*p*		OR (95% CI)	*p*
Males					
COVID-19 (2021 vs. 2017/2019)	1.01 (0.98–1.05)	0.4427		0.95 (0.89–1.02)	0.1557
Linear time (years from 2017)				1.02 (1.00–1.04)	0.0386
Females					
COVID-19 (2021 vs. 2017/2019)	1.50 (1.46–1.55)^*^	< 0.0001		0.99 (0.93–1.05)	0.6248
Linear time (years from 2017)				1.16 (1.14–1.18)^*^	< 0.0001
Males by age group					
Age group 13–15					
COVID-19 (2021 vs. 2017/2019)	0.96 (0.91–1.01)	0.0766		1.02 (0.93–1.13)	0.6511
Linear time (years from 2017)				0.98 (0.95–1.01)	0.1252
Age group 16–17					
COVID-19 (2021 vs. 2017/2019)	1.06 (1.01–1.12)^#^	0.0263		0.90 (0.81–0.99)	0.0392
Linear time (years from 2017)				1.06 (1.03–1.09)^§^	0.0003
Age group 18–20					
COVID-19 (2021 vs. 2017/2019)	1.06 (0.94–1.19)	0.3176		0.91 (0.72–1.15)	0.4483
Linear time (years from 2017)				1.05 (0.98–1.13)	0.1484
Females by age group					
Age group 13–15					
COVID-19 (2021 vs. 2017/2019)	1.51 (1.45–1.57)	< 0.0001		1.07 (0.98–1.17)	0.1601
Linear time (years from 2017)				1.13 (1.10–1.16)	< 0.0001
Age group 16–17					
COVID-19 (2021 vs. 2017/2019)	1.50 (1.44–1.57)	< 0.0001		0.95 (0.87–1.05)	0.2948
Linear time (years from 2017)				1.17 (1.14–1.21)	< 0.0001
Age group 18–20					
COVID-19 (2021 vs. 2017/2019)	1.48 (1.34–1.63)	< 0.0001		0.78 (0.63–0.96)^#^	0.0165
Linear time (years from 2017)				1.25 (1.17–1.34)^#^	< 0.0001

^*^ Effect significantly different from the corresponding effect in males, *p* < 0.0001

^#, §^ Effect significantly different from the corresponding effect in the youngest age group of same sex, ^#^*p* < 0.01, ^§^*p* < 0.001

**Table 4:** Effects of the COVID-19 pandemic (year 2021 vs. 2017/2019) and linear time on excessive Internet use adjusted for sociodemographic and mental health correlates by sex

	Model 1: COVID-19 only^b^			Model 2: COVID-19 + linear time^b^	
Subgroup / model	OR (95% CI)	*p*		OR (95% CI)	*p*
**Males**					
Unadjusted					
COVID-19 (2021 vs. 2017/2019)	1.01 (0.98–1.05)	0.4427		0.95 (0.89–1.02)	0.1557
Linear time (years from 2017)				1.02 (1.00–1.04)	0.0386
Adjusted 1 (sociodemographic factors)^a^					
COVID-19 (2021 vs. 2017/2019)	1.02 (0.98–1.06)	0.3080		0.98 (0.91–1.05)	0.5358
Linear time (years from 2017)				1.01 (0.99–1.04)	0.1967
Adjusted 2 (adj. 1 + loneliness)					
COVID-19 (2021 vs. 2017/2019)	0.99 (0.95–1.02)	0.4270		0.95 (0.88–1.02)	0.1364
Linear time (years from 2017)				1.01 (0.99–1.04)	0.2080
Adjusted 3 (adj. 2 + depression and anxiety)					
COVID-19 (2021 vs. 2017/2019)	0.96 (0.93–1.00)	0.0421		0.94 (0.87–1.01)	0.0804
Linear time (years from 2017)				1.01 (0.99–1.03)	0.3971
**Females**					
Unadjusted					
COVID-19 (2021 vs. 2017/2019)	1.50 (1.46–1.55)^*^	< 0.0001		0.99 (0.93–1.05)	0.6248
Linear time (years from 2017)				1.16 (1.14–1.18)^*^	< 0.0001
Adjusted 1 (sociodemographic factors)^a^					
COVID-19 (2021 vs. 2017/2019)	1.53 (1.48–1.57)^*^	< 0.0001		0.98 (0.92–1.04)	0.5038
Linear time (years from 2017)				1.17 (1.15–1.19)^*^	< 0.0001
Adjusted 2 (adj. 1 + loneliness)					
COVID-19 (2021 vs. 2017/2019)	1.46 (1.41–1.50)^*^	< 0.0001		0.96 (0.90–1.02)	0.1730
Linear time (years from 2017)				1.16 (1.14–1.18)^*^	< 0.0001
Adjusted 3 (adj. 2 + depression and anxiety)					
COVID-19 (2021 vs. 2017/2019)	1.34 (1.30–1.38)^*^	< 0.0001		0.90 (0.84–0.96)	0.0009
Linear time (years from 2017)				1.15 (1.13–1.18)^*^	< 0.0001

^a^ Including age group, family’s financial situation, living with both parents, origin, and region of Finland

^b^ In addition to the mentioned adjusted variables

^*^ Effect significantly different from the corresponding effect in males *p* < 0.0001

In the final phase of the analyses, the associations of loneliness, depression, and generalized anxiety with EIU were analyzed. This was done first separately for each study year in males and females, and then using year × mental health variable interaction terms in the sample comprising all study years to assess whether the associations in 2017 and 2019 were different from those observed in 2021 during the COVID-19 pandemic (Table 5).

**Table 5:** Effects of loneliness, depression, and generalized anxiety on excessive Internet use by sex and study year

	2017		2019		2021
Subgroup / model	OR (95% CI)		OR (95% CI)		OR (95% CI)
Males, adjusted^a^					
Loneliness	1.78 (1.59–2.00)		1.86 (1.68–2.05)		1.93 (1.78–2.09)
Depression	1.93 (1.75–2.13)		1.94 (1.78–2.11)		1.95 (1.80–2.10)
Generalized anxiety	2.14 (1.91–2.40)		2.54 (2.30–2.80)		2.27 (2.09–2.47)
Females, adjusted^a^					
Loneliness	1.47 (1.36–1.60)^§^		1.61 (1.51–1.71)^*^		1.76 (1.67–1.84)
Depression	2.05 (1.91–2.19)^§^		2.17 (2.06–2.30)^*^		2.34 (2.23–2.44)
Generalized anxiety	2.06 (1.92–2.21)^#^		2.27 (2.15–2.40)		2.26 (2.16–2.37)

^a^ Separate models for loneliness, depression, and generalized anxiety; all adjusted for age group, family’s financial situation, living with both parents, origin, and region of Finland

^*, #, §^ Effect significantly different from the corresponding effect in 2021 (using year × mental health variable interaction terms in the sample comprising all study years), ^*^p < 0.05, ^#^*p* < 0.01, ^§^*p* < 0.001

Note, all ORs are significant *p* < 0.0001

## Results

There were slight differences in the sex and age distributions between the years with proportions of females and younger participants increasing from 2017 to 2021 (Table 1). There were small increases in the prevalence of loneliness, depression, and anxiety between 2017 and 2019, but a sharp increase from 2019 to 2021.

The prevalence of EIU showed a gradually increasing pattern from 7.3% in 2017 to 9.9% in 2021 (Table 2). Among males, the prevalence was around 8% throughout the study period, while among females, there was a relatively strong increase from 6.8% in 2017 to 11.6% in 2021, when the difference compared to males was at its largest, 3.6 percentage points. Both in males and females the prevalence of EIU was highest among 13–15-year-olds throughout the study period, whereas the two older age groups did not differ from each other to any large extent (see also Supplementary Figure S1).

Among males, the effect of COVID-19 (year 2021) on the prevalence of EIU was non-significant (OR = 1.01, *p* > 0.4) compared to levels in pre-pandemic years, while among females, the effect was significant (OR = 1.50, *p* < 0.001) (Table 3, Model 1). However, when the linear trend between 2017 and 2021 was included in the model, the effect of the COVID-outbreak on the prevalence of EIU turned non-significant in females (OR = 0.99, *p* > 0.6), whereas the linear time was significant (OR = 1.16, *p* < 0.001) (Table 3, Model 2). The difference between sexes in the effects of COVID-19 (Model 1) and time (Model 2) were significant. These effects and sex differences in different models are highlighted in Supplementary Figure S2.

In males, there was a small effect of COVID-19 on EIU (OR = 1.06) in 16–17-year-olds and it was significantly different compared to the youngest age group (Table 3, Model 1). When linear time was included in the model, this effect turned negative (OR = 0.90), indicating that the prevalence of EIU in 2021 was smaller than expected given the underlying trend in EIU (Table 3, Model 2). Among the 16–17-year-olds, the linear time parameter indicated a significant (*p* < 0.001) gradual increase in EIU, and this effect was significantly different compared to the youngest age group (*p* < 0.001).

In females, there were no significant differences between the age groups in the effect of COVID-19 on EIU (Table 3, Model 1). When linear time was introduced to the models, the effect of COVID-19 turned negative in the oldest age group and the effect of time was more pronounced compared to the youngest age group (Table 3, Model 2).

The effects of adjusting variables on the parameter estimates of COVID-19 and time on EIU are presented in Table 4. Among males, the effects were small and negligible to begin with (unadjusted models) and remained largely unaffected in the adjusted models. Among females, the OR of COVID-19 on EIU, attenuated from 1.53 (in the model adjusted for sociodemographic factors) to 1.46 after introducing loneliness to the model and further to 1.34 after also adjusting also for depression and generalized anxiety, while remaining significant in all models (Table 4, Model 1). On the linear time parameter, the adjustments had practically no effect (Table 4, Model 2). In the final adjusted model in females, the effect of COVID-19 was negative (OR = 0.90, *p* < 0.001), indicating that the prevalence of EIU in 2021 was significantly lower than what would have been expected given the linear trend in EIU and controlling for related changes in the adjusting variables. Sex differences in the effects of COVID-19 (Model 1) and time (Model 2) remained highly significant in all adjusted models.

Associations of loneliness, depression, and generalized anxiety with EIU in each study year are presented in Table 5. All ORs were around 2.0, while somewhat smaller for loneliness, especially among females. Among males, the effects observed in 2021 did not differ significantly from those in 2017 or 2019. Among females, the effects in 2021 were significantly higher compared to the effects in 2017. Compared to the effects in 2019, the differences were smaller.

## Discussion

This study examined the prevalence of excessive Internet use (EIU) among Finnish young people in three large cross-sectional population-based samples between 2017 and 2021. There was a sex difference in the way the prevalence of EIU developed from 2017 to 2021, since increases were observed only among females and to the extent that EIU was more common in females in 2021 compared to males, while in 2017 the opposite had been the case. Changes in the prevalence were addressed with a special focus on the effects due to the COVID-19 pandemic. When the effect of COVID-19 was studied as a contrast between the year 2021 and the years 2017/2019 combined, there was a clear effect indicating a strong increase in the prevalence of EIU. This effect, however, was found only among females, whereas among males there were practically no changes in the EIU prevalence during the study period. Furthermore, when the linear time trend was included in the model, the COVID-19 effect also disappeared among females, suggesting that increases were perhaps not due to the COVID-19 pandemic, but rather due to a trend which began before the pandemic. These results are in contrast with some earlier studies suggesting an effect of COVID-19 on EIU. Adjusting the models with relevant mental health-related factors more heavily affected the effect of COVID-19 (i.e., the contrast between the years 2021 and 2017/2019), while not so much the linear trend. Among females, there was a slight intensification in the associations of loneliness, depression, and generalized anxiety with EIU, but the changes seemed more gradual during the study period, not solely attributable to the COVID-19 pandemic.

In this study, the prevalence estimates for EIU ranged between 7.3% and 9.9%, depending on the study year. These estimates seem moderate, as in many recent studies among adolescent and young adult populations, the prevalence rates have been considerably higher, even up to 30% and beyond [11, 12]. However, the prevalence rates in the present study are more in line with the pooled prevalence of 7.0% for IA in the meta-analysis by Pan et al. [8] for the total population (i.e., including adults). Furthermore, the prevalence rate of EIU in a study representative of Japanese adolescents was 7.9% [39] and a meta-analysis by Shao et al. [9] reported prevalence of 11% of IA among Chinese college students. It is typical that the prevalence rates of EIU or IA vary widely depending on sample characteristics and study designs and the larger prevalence rates in many instances are from convenience or otherwise unrepresentative samples [8, 10]. Different measures and selected cut-points to detect excessive use of Internet contribute to these differences as well [10]. For example, if we had used continuous EIUS-score with the 2.6 cutoff suggested by Škařupová et al. [28] in their quasi-validation study among 11- to 16-year-olds in 25 European countries, the prevalence of EIU would have been about twice as high as presented here. In the present study, a more symptoms-based approach was used to detect presence or increased risk of behavioral addiction [4, 31]. Nevertheless, these greatly varying prevalence estimates due to methodological issues, is a challenge for the field, and underline the urgent need for some standard, agreed-upon criteria to gauge different measures. In the meantime, studies using representative samples should be prioritized at least.

More importantly than the prevalence rates as such, the results of this study show a clear increase in them during the study period. Albeit the study period is too short to make strong claims about a trend, the findings are in line with recent studies suggesting increasing prevalence rates of IA [8, 9]. Most interestingly though, the present analyses indicated a marked sex difference in the way the prevalence rates changed between the years, in that the increases were observed only among females, while among males the trends were very flat. Furthermore, these sex-divergent trends resulted in a situation, where adolescent females reported higher prevalence of EIU than males in 2021. Typically, previous studies have reported higher prevalence rates for males than females [9, 13], while there are also studies where the opposite has been the case [39]. Thus, there might be happening a shift from male to female preponderance in EIU among Finnish adolescents. This pattern of findings seems understandable given the emphasized role of social media use among adolescents, especially females, combined with the recent findings that social media addiction is more common among them compared to males, while gaming disorder is more prevalent in males [14]. This would also further indicate that the more general categories of EIU and IA might mask some potentially relevant differences between sexes in the mechanisms and subtypes behind these addictive behaviors [14]. No large differences were observed between the age groups in the trends of EIU, while towards the end of the study period, the prevalence of EIU was largest among younger females.

One specific aim of the present study was to assess whether there were changes in the prevalence of EIU due to the COVID-19 pandemic. During the pandemic there were many reports among adolescents and young people suggesting increases in the prevalence of EIU, while majority of the studies were based on relatively small or unrepresentative samples and cross-sectional designs [11, 17]. In the present study, large, repeated population-based surveys from the pre-pandemic years 2017 and 2019 as well as from the spring of 2021 when Finland was facing the third wave of the pandemic, were available for the analysis and with the same measure of EIU. And the results did indeed show that the prevalence of EIU was clearly higher (again only in females) in 2021 comparing to the pre-pandemic levels, thus suggesting, and in line with the aforementioned studies, an effect of COVID-19 on EIU. However, the results also indicated, that this increase in 2021 could be totally explained by the increasing trend in the prevalence of EIU that started already during pre-pandemic years. This type of explanation would be in line with the studies suggesting increases in EIU already in pre-pandemic era [8, 9]. So, the answer to the question of the COVID-19 effect on EIU seems equivocal depending on the way pre-pandemic comparisons and developments are modelled. Coming rounds of data collections of the SHP study will shed further light on whether the increasing trend of EIU will continue or whether the increase between years 2019 and 2021 was a peak and then more likely related to the pandemic. Nevertheless, these analyses indicate the importance of careful modeling of the pre-pandemic developments and preferably using the same measures throughout, when claiming effects due to the COVID-19 pandemic.

The associations of EIU with loneliness, depression and anxiety were clear and in line with previous studies [3, 24]. While some studies have suggested that these associations might have been intensified during the pandemic [26], in the present study, only weak support for this was found. There were some indications among females that the associations between EIU and the studied mental health correlates were stronger in 2021 compared to the pre-pandemic years, but this intensification of associations seemed to have taken place to a large extent already during the pre-pandemic years. Furthermore, the changes in the associations were of relatively small magnitude and thus might also be chance findings.

Finally, the question was addressed whether the contemporaneous changes in loneliness, depression and anxiety would be related to and explain changes in the prevalence rates of EIU. There were clear increases in the prevalence rates of loneliness, depression, and anxiety in 2021 suggesting effects due to COVID-19 on these, as has been reported earlier from these data [40, 41]. And, when these measures were controlled in the analyses, they explained the modeled effect of COVID-19 on EIU to a considerable extent. However, they did not seem to explain the underlying increasing trend of EIU at all. This is interesting given the earlier studies showing increases in depression and anxiety during the 2010s among females [41, 42] and suggestions that these trends are linked to the increased smartphone and Internet use [15, 43]. While the mental health variables were correlated with EIU and their prevalence rates have been increasing during the study period (and earlier), in the light of the present analyses they are not likely causes behind the increasing trend of EIU.

### Strengths and limitations

One strength of the study is the large population-based sample with over 450,000 participants, good nationwide coverage, and generally high response rates. Biennial assessments from 2017 to 2021 enabled analyses of the effect of COVID-19 on EIU both as an increase compared to the pre-pandemic levels, but also as a deviation from the underlying trend in EIU. The data also enabled controlling the developments in EIU for a set of sociodemographic and mental health-related correlates that were all available for the whole study period. However, the study period is relatively short, and three measurements are rather few to make strong claims as to the more detailed or longer-term shape of the trend in EIU.

While the EIUS measure has shown good convergent validity in large European adolescent samples, it has not been validated against any clinical or diagnostic gold standard for Internet addiction or related disorders [28]. The measure, however, is based on a theoretical components model of behavioral addictions [4] and the category of EIU used here was based on criteria for certain symptoms presented [31], instead of some chosen cut point of the scale score.

When generalizing the results to the whole Finnish youth population, some caution is warranted, because those not attending school at the time of the study or who were unwilling to participate may be at greater risk for EIU or mental distress than those who took part in the study. Also, the sample coverage rate of students from vocational education institutions was considerably lower than students from other schools. As the data comprises three cross-sectional surveys, individual level changes cannot be assessed.

## Conclusions

There seems to be a sex difference in the way the prevalence of EIU has develop between years 2017 and 2021 among Finnish adolescents. In males there was no indication of increased prevalence of EIU during COVID-19 and among females, while the effect of COVID-19 was found, it was also suggested to be a product of a trend, beginning already before the COVID-19 pandemic. These results are in contrast with some earlier studies suggesting an effect of COVID-19 on EIU and highlight the importance of carefully considering the selected baseline reference points as well as underlying longer-term trends when claiming effects due to the COVID-19 pandemic. The associations with mental health indicators indicate the public mental health concerns justified relating to EIU among adolescents. Monitoring adolescent mental health and EIU is warranted in the aftermath of the COVID-19 pandemic, while keeping in mind the trends originating in the pre-pandemic years.

## Electronic supplementary material

Below is the link to the electronic supplementary material.

Supplementary Material 1
