# Supplementary material for: Excessive internet use among Finnish young people between 2017 and 2021 and the effect of COVID-19
Source: Soc Psychiatry Psychiatr Epidemiol. 2024 Jul 10;59(12):2291–301. doi: 10.1007/s00127-024-02723-0 (PMC11522120; doi:10.1007/s00127-024-02723-0)
Supplement: Supplementary file 1 — Supplementary Material 1 [file 127_2024_2723_MOESM1_ESM.pdf]

Supplementary Material for “Excessive Internet use among Finnish young people between 2017 and 2021 and the effect of COVID-19”

Journal: Social Psychiatry and Psychiatric Epidemiology

Author: Olli Kiviruusu, Finnish Institute for Health and Welfare, e-mail olli.kiviruusu@thl.fi

COVID-19 situation and the related restrictions in Finland in spring 2021

In spring 2021, the SHP study was carried out at a time when Finland was facing the third wave of the pandemic. There had been many restrictions in effect for all age groups since the spring of 2020, including gatherings of more than 10 people being prohibited, a recommendation for physical distancing, recommendations for remote work for adults, and suspension of collective leisure-time activities. School closures were implemented as part of lockdown efforts and at the time of the study, most adolescents from upper secondary schools had spent a large part of the 2020–2021 school year in remote education, but recently returned to in-person learning. Students in comprehensive schools, however, had spent almost the entire school year 2020–2021 in on-site learning.

Details and the coding of the measures

Excessive Internet use (EIU). EIU was measured with the 5-item version of the Excessive Internet Use Scale (EIUS) [28]. The items cover five components of behavioral addictions, i.e., cognitive and behavioral salience, tolerance, withdrawal symptoms, conflicts with other important activities and social relationships, and relapses when trying to control the behavior [4, 29]. The statements were: “I have tried spending less time online, but I have failed” (relapse); “I should spend more time with my family, friends or homework, but I spend all my time online” (conflict); “I have found that I was online even though I did not really feel like it” (tolerance); “I have felt anxious when I do not get online” (withdrawal symptoms); “I have failed to eat or sleep because of being online” (salience). In the instruction stated before the items, a broad definition of being on the Internet/online was given to include various leisure time activities (gaming, social media, etc.) and different devices used to access the Internet (phone, tablet, computer, etc.). The items were answered on a four-point scale (1 – “never”; 2 – “not very often”; 3 – “fairly often”; 4 – “very often”) and the answers “fairly often” and “often” indicated a present symptom. The condition where the conflict symptom and at least three other symptoms were present was considered to

indicate an increased risk of addictive behaviors [30, 31] labeled here as “excessive Internet use” (EIU) and was the outcome of the present study.

Loneliness. Loneliness was asked with a simple question “Do you ever feel lonely?" From the five answer categories "never,” “very rarely,” “sometimes,” “fairly often,” and “all the time”, a dichotomous (yes/no) variable for loneliness was coded: yes (“fairly often” or “all the time”) vs. no (otherwise).

Depression. Depression was measured with the Patient Health Questionnaire-2 (PHQ-2) [32]. It is a 2-item self-reported screen shown to be a reliable tool among adolescents and adults to detect depression [32, 33]. The items (scale 0–3) cover low mood and anhedonia, two key symptoms of DSM-5 major depressive disorder [6]. The sum score (range 0–6) was dichotomized to depression (3 or more points) vs. no depression (<3) [32, 33].

Anxiety. Anxiety was assessed using the 7-item Generalized Anxiety Disorder Scale (GAD-7) [34]. The GAD-7 has proved to be a reliable and valid instrument for measuring self-reported generalized anxiety in the general population among adults [35] and adolescents [36]. The items (scale 0–3), such as "worrying too much about different things" and "not being able to stop or control worrying", cover the most prominent diagnostic features of the DSM-5 generalized anxiety disorder [6]. When calculating the sum score, two missing items were allowed and replaced by the mean of the respondent’s other items (9 199, 2.0%). The sum score (range 0–21) was dichotomized using the cut-off of 10 points or more to indicate cases with moderate to severe self-reported generalized anxiety symptoms [34], hereafter “generalized anxiety”.

Sex and age. The respondents reported their sex (male or female). Age (truncated to whole years) was based on year and month of birth reported by respondents. For 2,466 (0.5%) cases, missing age was replaced by the mode age in the grade there were in. In addition, there were 1,209 (0.3%) cases reporting unreliable age—too young or too old for the given school grade (e.g., less than 14 years old in upper secondary education). These were coded as missing information and thereby excluded from the analyses. The SHP is conducted between March and May when most of the 8th graders are 14 to 15 years old (98.5 % in the present sample) and 9th graders are 15 to 16 years old (98.2 %), while 1st-year students in general upper secondary level and vocational education institutions are mostly 16 to 17 years old (94.0 %), and 2nd-year students 17 to 18 years old (95.5 %). The mean age of the total sample was 15.8 (SD = 1.28) years. For the analyses, the following three age groups were formed: 13–15-year-olds, 16–17-year-olds and 18–20-year-olds (Table 1). Of note, there were only 255 respondents aged 13 years old in the age group of 13–15-year-olds.

Control variables. The following four sociodemographic factors were used as control or adjusting variables in the analyses. Family's financial situation were measured using the question: "How would you rate your family's financial situation?" with the response options "very good," "fairly good," "moderate," "fairly poor," and "very poor". For the analyses, a three-category variable was formed: "good" (very/fairly good), "moderate" (moderate), and "poor" (fairly/very poor). Participants reported whether they lived in a household with both parents (yes/no). Origin was based on questions about the country of birth of the respondent and the parents and was classified into four categories: 1) Finnish-born parents (respondent and parents born in Finland), 2) multicultural family (respondent and one parent born in Finland, one parent born abroad), 3) second-generation immigrant (respondent born in Finland, parents born abroad), and 4) first-generation immigrant (respondent and parents born abroad). The geographical region was based on the seven Regional State Administrative Agencies (Table 1). These agencies also decided and administered many of the COVID-19-related restrictions, including restrictions on schools, leisure time activities, and hobbies.

Note. Reference numbers in the above text refer to the list of references of the main article.

Exclusions due to implausible responding

In the analyses, additional 1,593 (0.4%) cases were excluded due to implausible (or unreliable) responding, namely reporting in three questions on functional limitations that they were completely unable to see, hear, and walk, which would mean that they would be highly unlikely to attend the unspecialized schools that participate in the SHP or to be able to respond to the questionnaire if they actually had these limitations [*1].

Reference

*1. Kaltiala-Heino R, Lindberg N (2019) Gender identities in adolescent population: methodological issues and prevalence across age groups. Eur Psychiatry 55:61–66. https://doi.org/10.1016/j.eurpsy.2018.09.003

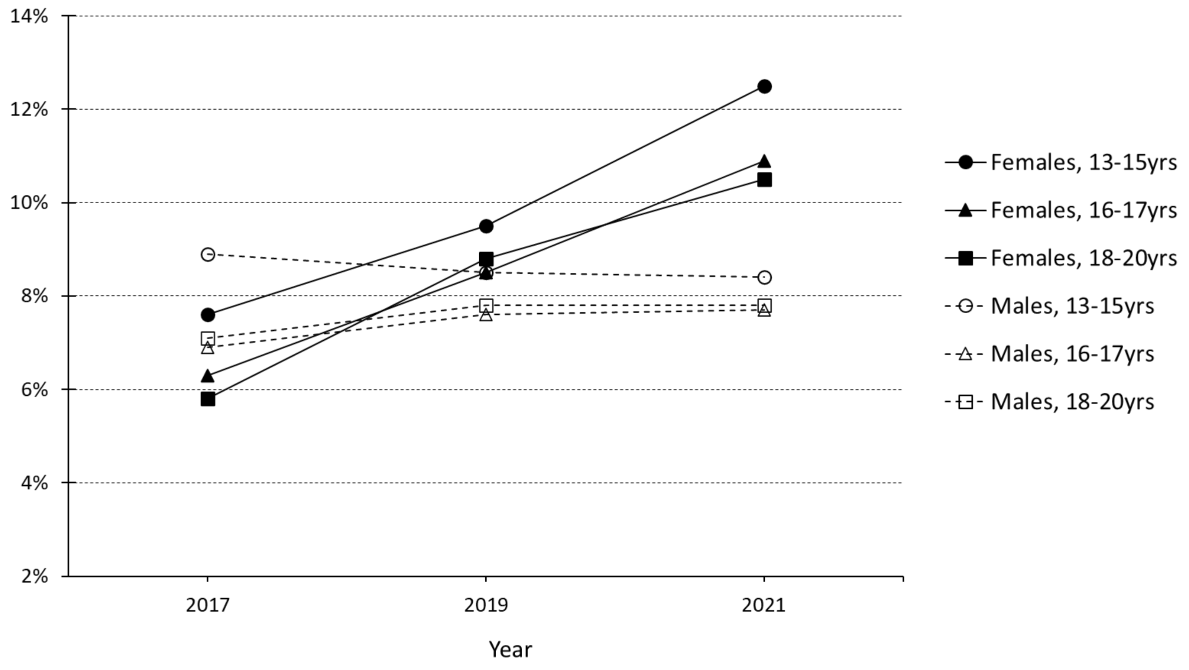

Supplementary Figure S1

Percentages of those with excessive Internet use (EIU) among Finnish young people in 2017–2021 by sex and age group.

A)

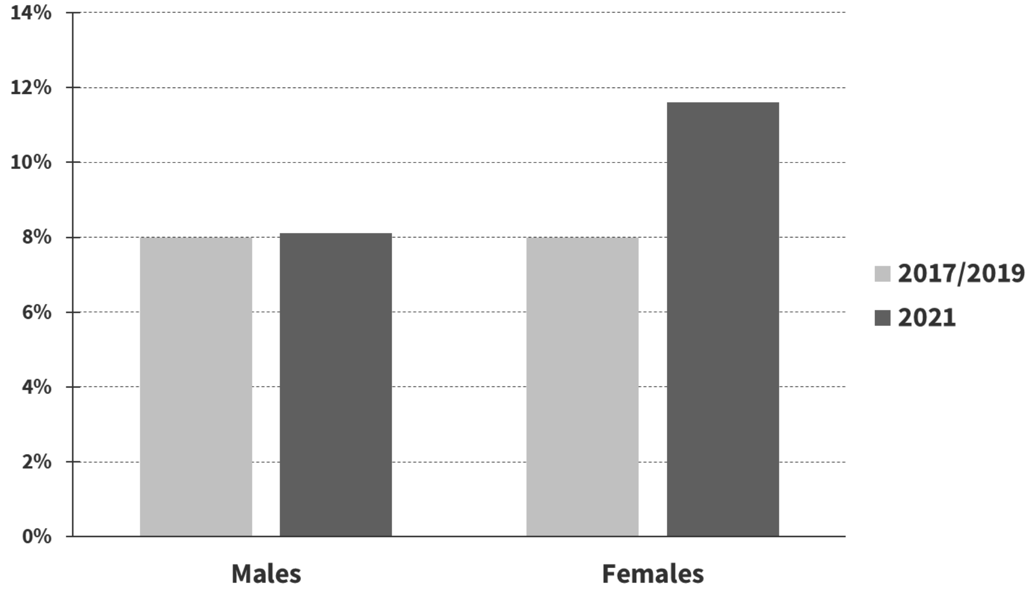

B)

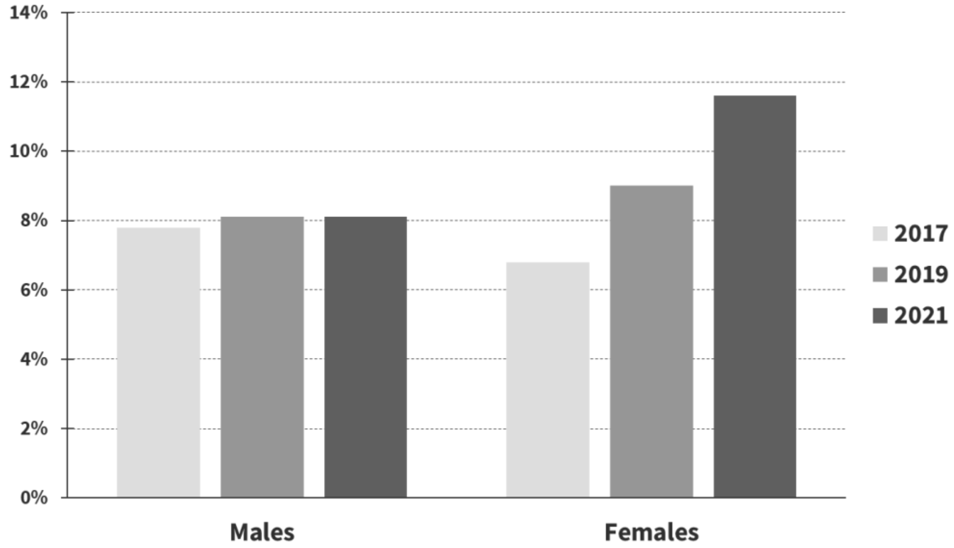

Supplementary Figure S2

Percentages of those with excessive Internet use (EIU) among Finnish young people by sex. A) pre-pandemic years 2017/2019 combined vs. 2021; B) years 2017, 2019, and 2021 separately.
